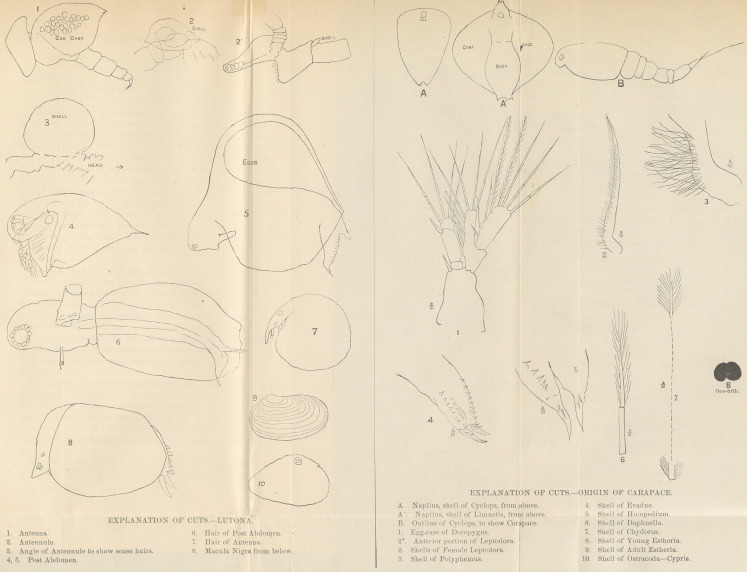# Notes on Crustacea in Chicago Water Supply, with Some Remarks on the Formation of the Carapace

**Published:** 1881-12

**Authors:** E. A. Birge

**Affiliations:** Professor of Zoölogy, University of Wisconsin, Madison, Wis.


### ARTICLE III.

### NOTES ON CRUSTACEA IN CHICAGO WATER SUPPLY, WITH REMARKS ON THE FORMATION OF THE CARAPACE. By E. A. BIRGE, Professor of Zoölogy, University of Wisconsin, Madison, Wis.

Through the kindness of President B. W. Thomas, I have from time to time received valuable specimens of the animal and vegetable life of the Chicago water supply, a subject to which he has devoted much labor. In these specimens I have observed the following species of Cladocera, the group of Crustacea in which I am especially interested.

Sididæ. { Sida crystallina—O. F. Müller.

Latona setifera (?)—O. F. Müller.

Daphnidæ. { Daphnia pulex—De Geer.

" galeata (junior) (?)

Bosminidæ. — Bosminia longirostris (?)

- Camptocercus macrurus—O. F. M.

- Pleuroxus insculptus—Birge.

- " denticulatus—Birge.

- Chydorus sphæricus—O. F. M.

- Alona (?) two species unidentified.

All these species, except the second, are abundantly distributed in this country and elsewhere. They are what might be expected in any water derived from lakes, and do not argue any special impurity in the water. Indeed, with the doubtful exception of Alona, they are all species preferring the clearer waters. Alona is a genus of very various habits, some species being decidedly limicolous, while others haunt weedy but clear water, and others still may be found in places quite free of weeds. The list is thus, on the whole, conspicuous for the absence of dirt-loving Cladocera. The absence of some well-known species may occasion some surprise. None of the very abundant forms are present except Chydorus. One would have looked for some Simocephalus or Ceriodaphnia, and no doubt these will be found in due time.

I have at hand no very full description of Latona setifera, O. F. M., with which to compare the specimens sent me from Chicago. The two descriptions which I have—Leydig's and P. E. Müller's—are not in complete accord. Still, I think that our specimens belong to that species. They had been mounted, and so had been flattened, and unfortunately, in every case were vertically compressed. So delicate were they, that I could not get them flattened in the other direction, and must content myself with some notes and rude sketches of some of the details of structure.

The animal is everywhere exceedingly rare. It has been seen in Germany, in Sweden and in Denmark, in Europe, by Müller Sars and P. E. Müller, respectively, and in this country by Mr. Edward Burgess, of the Boston Society of Natural History, in Cochituate water, and now by Mr. Thomas in Chicago. Other observers have probably met with it, but I have no record of the fact.

The size of the specimens is from two to two and a half mm. in length. The lower edge of the valves is armed with numerous long setæ, from which the name of the species is derived. Leydig (Naturgeschichte der Daphniden) says that the anterior and posterior edges only of the valves are thus armed, but as he had not seen the species himself, this may easily be an error. Between each of the large setæ are three or four very small spines. Fornices are present. The rostrum forms a broad plate projecting over the base of the rather small labrum. The antennules are long, and covered with sense-hairs to the end. I find no trace of the "setæ sensuales obtusæ" described by P. E. Müller (Danmarks Cladocera, Pl. VI, fig. 22), and figured by him on the inside of the antennule, near the base. (See my figs. 2 and 3). The antenna (fig. 1) afford one of the most striking peculiarities of the animal. They appear three-branched, but in reality have but two branches, like those of all Cladocera. The basal joint of the two-jointed branch is projected out between the two branches, so as to look like a third division. The joints of the three-jointed branch bear respectively 4–0–0 setæ; of the other branch 8–9 setæ respectively; spines 0, 1, 0 and 0, 0, and one on the basal joint. These setæ are plumose, especially the terminal joint (fig. 7).

Both maxillæ seem to be present. This fact was noted by Claus (Zeitschrift für Wissenschaft. Zoölogie, Vol. XXVII) in Sida, but I have been entirely unable to confirm his observations by my own on American specimens. Here, however, both seem to be functional.

As in Sida, there are six pairs of legs, and the post-abdomen closely resembles that of Sida. It has eight abdominal teeth in each row. The terminal claws are provided with two spines, and a row of very fine teeth on each edge (figs. 4, 5). The abdominal setæ are two-jointed, and the terminal joint bears long, fine hairs (fig. 6).

The macula nigra is double (fig. 8).

*On the Origin of the Carapace in Crustacea.*—In his Anatomy of Invertebrated Animals (p. 315), and again in his Crayfish (p. 449), Prof. Huxley brings forward—not for the first time—his theory of the decapod carapace. Briefly stated, it is this: "The carapace corresponds with the terga and tergal halves of the pleura of all the somites, which are thus reflected into it, and these include, without exception, all from the last thoracic to the ophthalmic." The ordinary theory is well known. It is that of Milne Edwards, that the post-mandibular part of the carapace is a reflection of the tergum and pleura of the mandibular segment.

It may be questioned whether a form bearing only three segments can be the primitive form of crustacea. Whether the nauplius, however, is or is not such a form, makes little difference, since all groups of crustacea pass into the nauplius stage, and their carapace is derived in some way from the nauplius-shell. This nauplius-carapace, then, we may call the *primitive carapace* (figs. A, $A^1$). It is an oval, unsegmented shield, hardest on the back, where it serves both for protection and for attachment of the muscles which move the appendages. These are now three, antennule, antenna, mandible. This carapace may barely cover the back, as in Peltogaster and many other forms, or, as in Limnetis, it may extend broadly out on the sides.

In no adult form is the number of segments so small as three, and in all forms which pass through the nauplius stage after leaving the egg, more or fewer of the segments which appear as development goes on, are anchylosed to the three already present. The two maxillary segments are always thus united, and others may be, up to the number of eight. Thus a sort of carapace may be found covering the first five segments at least. This carapace may, when developed, take one of two general forms, which we may name respectively the *copepod-form* and the *phyllopod-form*.

If the development of a form like Cyclops among the Copepoda be watched, it will be seen that the shape of the body and the direction in which the appendages point is gradually altered, as well as the shape of the appendages and the number of segments. The body, at first relatively flat and thin, becomes horizontally compressed, and the appendages, from pointing outward, are directed downward. The carapace also undergoes modification. It is extended downward into pleura which protect the bases of the legs, and enlarged by the addition to it of new segments, in Cyclops three in addition to those already present. In other Copepoda more or fewer segments may be added, but in all cases the result is the same—a firm anterior shield is formed by the coalescence of the skeletons of the anterior segments, and this is extended downward into pleura to protect the appendages (fig. B). This form is found not only in Copepoda, but in the anterior shield of Trilobita, in Pœcilopoda, in the heads of Amphipoda and Isopoda, and in Arachnida and Insects. This form of carapace plainly agrees with Prof. Huxley's definition.

Very different is the origin of the other form. The nauplius larva of Limnetis gives us a hint of its source. The phyllopod form of carapace arises from the backward extension of the nauplius-carapace, covering the segments which are developed behind the mandibles. Our Cladocera show it in a well developed form, a large bivalve shell, covering body and legs, but free from both (figs. 5, 6, 7). In other forms it is more extended, enveloping not only body, but head as well. Such is the case in the Ostracoda, in Estheria, Limnetis and allied forms (figs. 10, 9). In these cases it always, I think, arises from the mandibular segment, although such is not necessarily its origin.

Before passing to consider to which form the decapod-carapace is allied, let us cast a glance at the possible origin of this form of carapace. In Notopterophorus, a copepod, we find the dorsal parts of the segments prolonged into a sort of crest, and in Doropygus we find the last thoracic segment expanded, and used as an egg-case (fig. 1). This case is of course homologous in a broad way to the two cases in which Cyclops carries its eggs, but here we have the skeleton more obviously made use of as a case.

Now, when we come to the Cladocera, we find the bivalve shell made use of as an egg-case, and in certain forms—the *Gymnomera* of Sars, including the genera Leptodora, Polyphemus and Bythotrephes—as such a case only. In these the carapace is rudimentary (figs. 2, $2^1$, 3). Leptodora has a long segmented abdomen; only the females have a small shell, capable of holding two eggs; the males and young females are shell-less. Polyphemus has a small but permanent shell. Evadne has a much larger carapace (fig. 4), but one from which the legs are still free. Holopedium (fig. 5) has the regular phyllopod form, but has the egg-case still as a prominent feature. In Daphnella (fig. 6), we get the ordinary form.

In Chydorus we find the carapace beginning to encroach on the head, and this feature carried to extremes in Ostracoda and Limnadidæ (figs. 7, 8, 9, 10).

Now, is this series, from Daphnella to Leptodora, given in the direct or inverse order? Have we here a direct or a retrograde metamorphosis? Is the carapace a developed egg-case, or the egg-case a rudimentary carapace? The question is too large to be answered fully at present, but one or two considerations may be urged to show that the carapace develops from the egg-case. The Cladocera are a very old group, showing all sorts of cross-affinities and retention of primitive features where they would least be expected. Such is the curious modification of the primitive antennule—undoubtedly like that of Latona—seen in the Bosminidæ and in the males of Ceriodaphnia, and other such features might be adduced. Two groups are remarkable for retaining primitive structures—the Sididæ and Gymnomera—and the position turns on the decision as to which of them most nearly represents the primitive form. From the structure of the antennules, of the antennæ especially, and of the legs, I conclude that the latter represent more nearly the "Ur-cladocera," and this conclusion is strengthened by the fact that Leptodora alone of the Cladocera has a free nauplius form. I believe that the phyllopod carapace originated as an egg-case.

To which form, then, is the decapod carapace allied? Whatever be the answer for Astacus, it seems tolerably plain for the rest of the Podophthalmata. Passing over Schizopods, a glance at the development of Peneus will show that its carapace belongs to the phyllopod type. It is free from the body behind the mandibles, and remains so for a very considerable time. So, too, the carapace of Squilla remains permanently free. The same is true for all decapods which pass through the zoëa stage. The zoëa-carapace is plainly of the phyllopod type (to which group, indeed, the zoëa is allied in many ways), and the carapace of the adult is derived from that, either directly, or indirectly, by means of the megalops stage. In no case of decapods which undergo a metamorphosis, can the carapace be referred to the copepod type.

What modifications this carapace may undergo in so modified a development as that of Astacus, may be a subject for further discussion. But plainly the inference which follows from Prof. Huxley's treatment of the subject in his Invertebrates, is incorrect. The decapod carapace in general conforms to the phyllopod type, not to that of the Copepoda.

I do not think Prof. Huxley's position tenable for the crayfish itself. On this point I speak with great diffidence, since I have never investigated the embryological development of the crayfish for myself. In studying that of Panopæus, I observed that the carapace, soon after its first appearance, apparently belonged to the segments as far back as the first maxillipedes. Subsequent changes showed that this was not the case, but that the still relatively undifferentiated condition of the blastoderm gave rise to the appearance. After hatching, the true relations of the carapace plainly appeared. In the passage from the zoëa to the megalops stage, the carapace, once free, became attached to the hinder thoracic segments. Now, my own belief is that in the crayfish, since it leaves the egg in a post-megalops stage, has syncopated this process of development and subsequent adhesion, and that consequently the appearance is that the carapace belongs to fourteen segments, instead of three. I have, however, no direct proof of this theory. It is only a "guess at truth," and I offer it merely as such.

## Figures and Tables

**Figure f1:**